# Effect of high-intensity interval training on clinical outcomes in lung cancer patients undergoing surgery: a meta-analysis based on randomized controlled trials

**DOI:** 10.3389/fmed.2026.1868572

**Published:** 2026-07-10

**Authors:** Dongjun Li, Hongxia Chen, Qianyu Zuo, Fulong Lv, Kuanhong Shen, Mao Sun

**Affiliations:** 1 Department of Rehabilitation Medicine, An Shun City People’s Hospital, Anshun, China; 2 Department of Thoracic, Breast and Thyroid Surgery, An Shun City People’s Hospital, Anshun, China; 3 Department of Pharmacy Laboratory, An Shun City People’s Hospital, Anshun, China

**Keywords:** clinical outcomes, high-intensity interval training, lung cancer, meta-analysis, surgery

## Abstract

**Purpose:**

This study aimed to clarify the impact of high-intensity interval training (HIIT) on clinical outcomes in lung cancer patients undergoing surgery based on available randomized controlled trials (RCTs).

**Methods:**

PubMed, Web of Science (WoS), the Cochrane Library, and CNKI databases were searched from their inception to 3 November 2025. The primary outcomes were postoperative complications such as pulmonary infection and atelectasis. The secondary outcomes included the postoperative length of stay (LOS), exercise capacity, pulmonary function, quality of life, respiratory muscle strength, and emotional outcomes.

**Results:**

A total of 11 RCTs were included in the study. Pooled results revealed that HIIT significantly reduced the risk of pulmonary infection (odds ratio [OR] = 0.38, *p* = 0.002) and atelectasis (OR = 0.30, *p <* 0.001). In addition, HIIT significantly improved exercise capacity, as evidenced by increases in peak oxygen uptake (MD) = 4.31 mL/kg/min, *p <* 0.001; MD = 0.24 L/min, *p* = 0.01) and 6-min walk distance (MD = 44.50 m, *p* = 0.04). HIIT also enhanced pulmonary function, with significant improvements in forced expiratory volume in one second (MD = 0.13 L, *p* = 0.03), forced vital capacity (MD = 0.19 L, *p <* 0.001), and maximum voluntary ventilation (MD = 3.19 L/min, *p* = 0.002; MD = 3.45%, *p* = 0.04). Furthermore, quality of life improved as assessed by the SF-36 (overall score: MD = 8.32, *p <* 0.001; physical component: MD = 7.04, *p <* 0.001), and emotional status improved, reflected by decreased SAS scores (MD = −6.90, *p <* 0.001) and FoP-Q-SF scores (MD = −5.12, *p* = 0.002).

**Conclusion:**

Based on available evidence, HIIT may improve clinical outcomes among surgical lung cancer patients, such as decreasing risk of postoperative pulmonary infection and atelectasis and increasing pulmonary function, highlighting its potential clinical application in patients with lung cancer undergoing surgery.

## Introduction

Lung cancer remains the leading cause of cancer-related mortality worldwide, and surgical resection is still the mainstay of curative treatment for patients with early-stage disease (1, 2). Despite remarkable progress in surgical techniques and perioperative management, postoperative morbidity remains substantial, and optimizing perioperative care is essential to improve patient outcomes (3).

Patients undergoing lung cancer surgery are susceptible to a variety of adverse clinical outcomes, including postoperative pulmonary complications, prolonged hospitalization, and decreased physical performance and quality of life (4, 5). These complications are more frequent in high-risk patients, such as those of advanced age, with poor baseline pulmonary function, or with comorbidities (6). Therefore, perioperative rehabilitation strategies that enhance respiratory function, cardiopulmonary endurance, and recovery have gained increasing attention in thoracic surgery (7).

High-intensity interval training (HIIT), characterized by short bursts of vigorous exercise alternating with recovery intervals, has emerged as an efficient and effective exercise modality (8). Evidence from cardiac, abdominal, and orthopedic surgery has shown that HIIT can improve cardiorespiratory fitness, reduce postoperative complications, and accelerate functional recovery (9–11). However, the effects of HIIT on clinical outcomes in patients undergoing lung cancer surgery remain unclear. Recent systematic reviews have demonstrated that exercise-based rehabilitation may improve postoperative recovery and functional outcomes in patients with non-small cell lung cancer (12). However, these studies primarily evaluated general exercise interventions, and the specific role of HIIT has not been systematically clarified.

Therefore, this meta-analysis aimed to systematically evaluate the impact of HIIT on postoperative complications, functional recovery, and quality of life in lung cancer patients undergoing surgery, and to provide evidence for its clinical application in perioperative rehabilitation.

## Methods

This meta-analysis was conducted in accordance with the 2020 Preferred Reporting Items for Systematic reviews and Meta-Analyses (PRISMA) guidelines (13).

### Literature search

PubMed, Web of Science, the Cochrane Library, and CNKI databases were searched from their inception to 3 November 2025 with these terms: high-intensity interval training, HIIT, lung, pulmonary, tumor, cancer, neoplasm, and carcinoma. Detailed search strategies in the above databases are presented in Supplementary File 1. MeSH terms and free text terms were applied during the search process.

### Inclusion criteria

Studies that met these criteria were included: a. patients were diagnosed with primary lung cancer and underwent surgery; b. studies with a RCT design; c. patients were randomly divided into the HIIT group or control group, and studies were eligible if HIIT constituted the core component of the intervention, even when additional rehabilitation elements (e.g., breathing exercises or resistance training) were provided as part of routine perioperative care; d. at least one clinical outcome was reported, and all clinical outcomes, including postoperative complications, exercise capacity, pulmonary function, quality of life, respiratory muscle strength, and emotional outcomes, were defined and assessed according to internationally recognized guidelines or standardized criteria. For example, postoperative complications such as pulmonary infection, atelectasis, and arrhythmia were determined based on established clinical definitions, and grade 2 + complications were classified according to the Clavien–Dindo grading system; e. enough data were provided for the pooled analysis; f. articles were published in English or Chinese; and g. full texts were available.

### Exclusion criteria

Studies that met these criteria were excluded: a. confounding interventions were involved; b. duplicate or overlapping data; c. reviews, case reports, meeting conferences, or animal studies.

### Data extraction

The following information was collected from the included RCTs: the first author, year, country, number of patients in the HIIT group and control group, intervention period (pre-operation or post-operation), endpoints, and mean difference (MD) with standard deviation (SD), and odds ratio (OR) with 95% confidence interval (CI).

In our meta-analysis, the primary outcomes were postoperative complications, including pulmonary infection, atelectasis, arrhythmia, 30-day mortality, respiratory failure, cardiovascular complications, grade 2 + complications, cardiopulmonary complications, and bronchopleural fistula.

The secondary outcomes consisted of postoperative length of stay; exercise capacity including the peak oxygen uptake and 6-min walk distance (6MWD); pulmonary function including the forced expiratory volume in 1 s (FEV1), forced vital capacity (FVC), and maximum voluntary ventilation (MVV); quality of life evaluated by the Medical Outcomes Study Short-Form 36 general health survey (SF-36) and Lung Cancer subscale of the European Organization for Research and Treatment of Cancer, Quality of Life Questionnaire Core 30 (EORTC LC13); respiratory muscle strength including the maximal expiratory pressure (MIP) and maximal expiratory pressure (MEP); and emotional outcomes assessed by the Self-rating Anxiety Scale (SAS) and Fear of Progression Questionnaire-Short Form (FoP-Q-SF).

### Methodological quality assessment

The methodological quality of the included randomized controlled trials was assessed using the Cochrane risk-of-bias tool in accordance with the guidelines in the Cochrane Handbook for Systematic Reviews of Interventions. The following domains were evaluated: random sequence generation, allocation concealment, blinding of participants and personnel, blinding of outcome assessment, incomplete outcome data, selective reporting, and other potential sources of bias.

Study screening, data extraction, and quality assessment were performed independently by two authors. Any disagreements were resolved through discussion, and if necessary, by consultation with a third author.

### Statistical analysis

All statistical analyses were conducted using RevMan version 5.3 software. Continuous outcomes were analyzed as changes from baseline to post-intervention values whenever possible. When change scores were not directly reported, they were calculated from baseline and final values. If the standard deviations (SDs) of change scores were not available, they were estimated using the formula recommended in the Cochrane Handbook, assuming a conservative correlation coefficient (r = 0.5) between baseline and follow-up measurements.

For studies reporting medians and ranges, means and SDs were estimated using the method described by Hozo et al. (14), which assumes an approximately normal distribution of the data.

Heterogeneity among studies was assessed using the I^2^ statistic and the Q test. In addition to statistical heterogeneity (I^2^ > 50% and/or *p* < 0.10), clinical and methodological heterogeneities—such as differences in intervention timing, training protocols, and surgical approaches—were also considered when selecting the meta-analytic model. A random-effects model was used when substantial heterogeneity was present or when clinical diversity was evident; otherwise, a fixed-effect model was used (15, 16).

Continuous outcomes were summarized as MDs with 95% CIs, and dichotomous outcomes were expressed as ORs with 95% CIs. A two-sided *p*-value of < 0.05 was considered statistically significant.

Certainty of evidence was assessed using the Grading of Recommendations Assessment, Development and Evaluation (GRADE) approach. The certainty of evidence for each outcome was evaluated across five domains: risk of bias, inconsistency, indirectness, imprecision, and publication bias. As all included studies were randomized controlled trials, the initial certainty was rated as high and was downgraded as appropriate according to the GRADE criteria.

## Results

### Literature search

**Figure 1:**
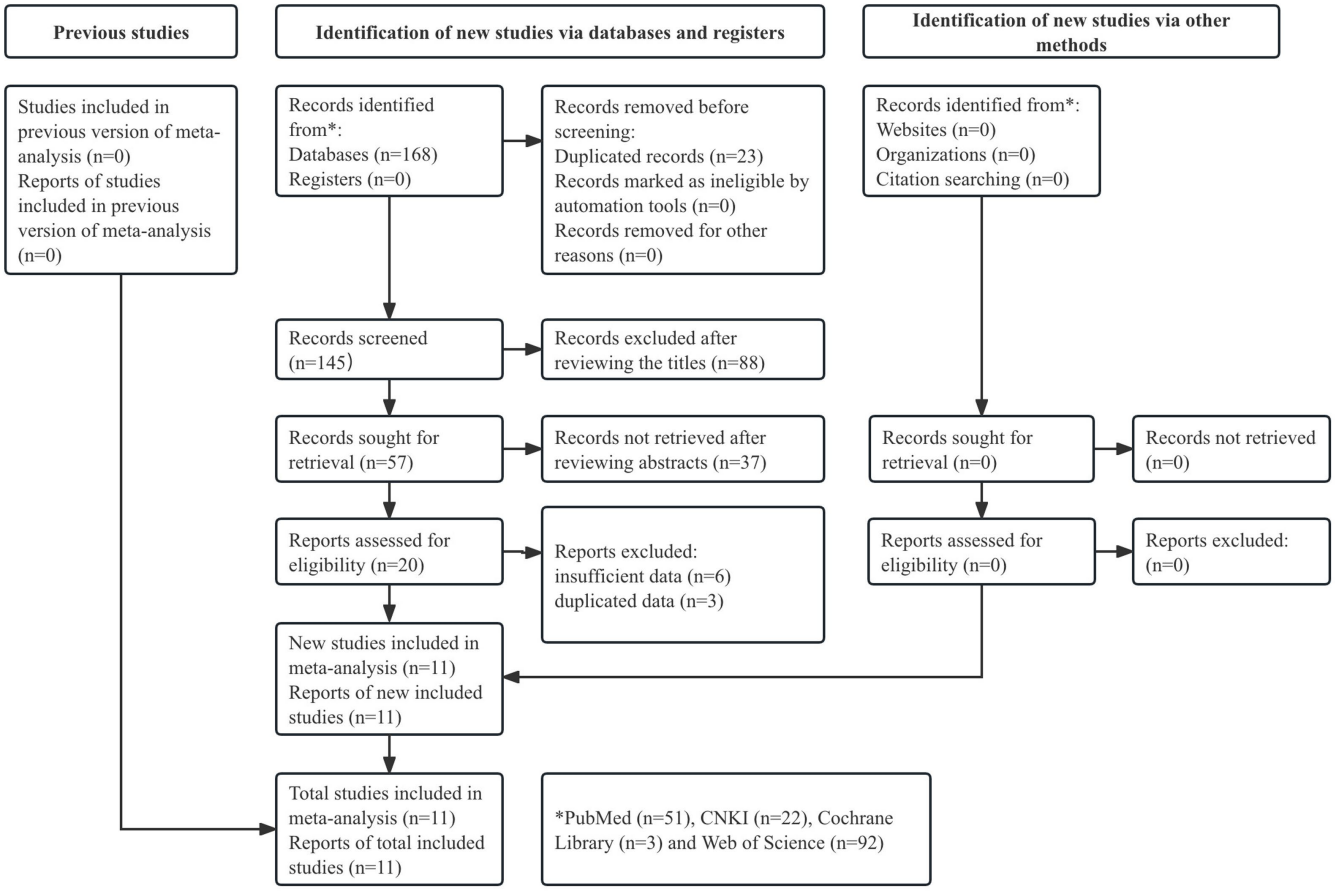
PRISMA flow diagram of this meta-analysis.

Figure 1 displays the literature search and selection process. A total of 168 records were identified from four databases, and 23 duplicate records were removed. After reviewing titles, abstracts, and full texts, 133 publications were excluded. Eventually, 11 studies were included in the final analysis (17–28).

### Basic characteristics

**Figure 2:**
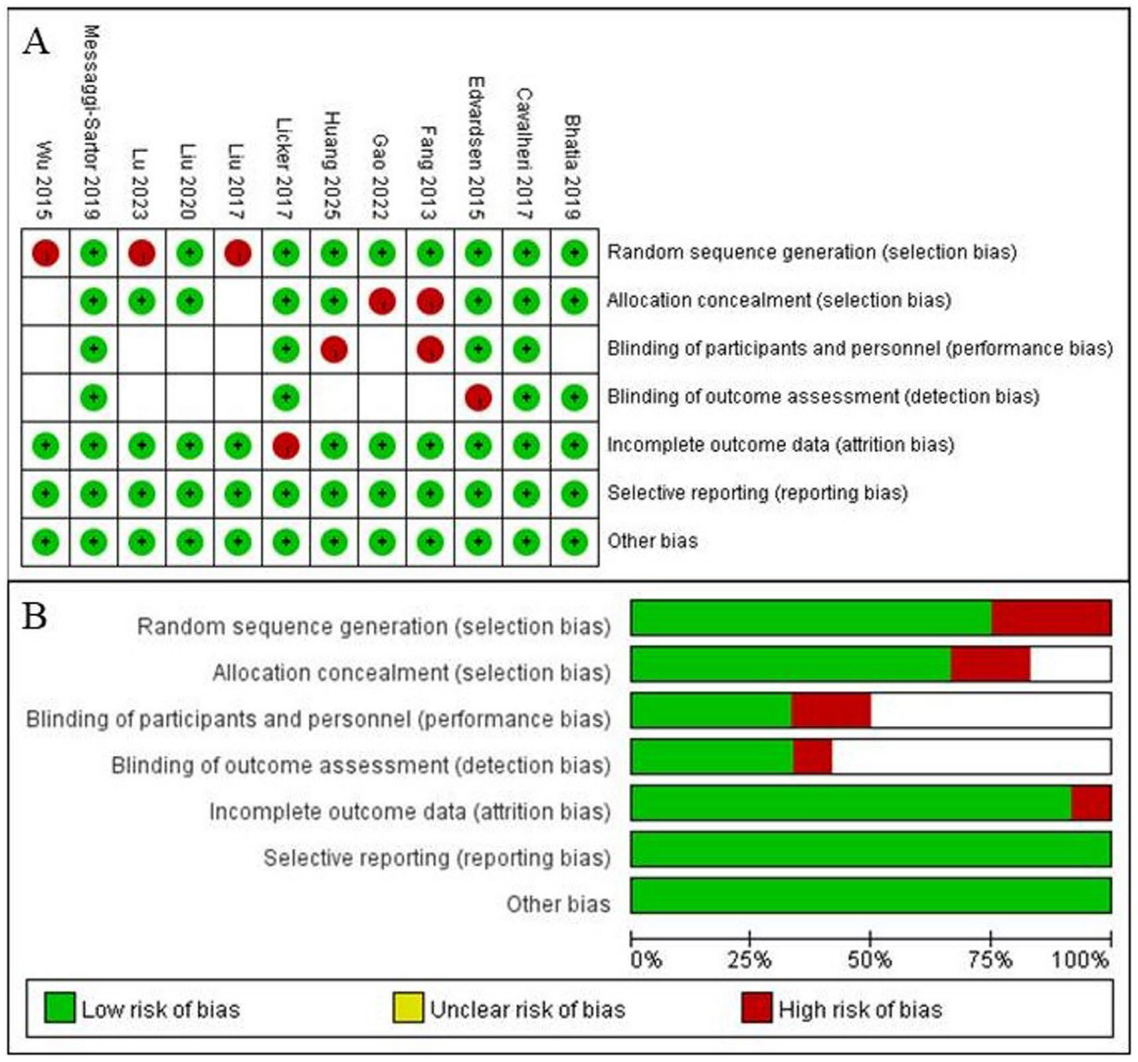
Bias risk of the included studies. **(A)** Risk of bias summary and **(B)** risk of bias graph.

Among the 11 included RCTs, 898 surgical lung cancer patients were included, with 444 patients in the HIIT group and 454 in the control group. The majority of studies were from China (7/11) and focused on preoperative HIIT intervention (7/11). The majority of studies implemented HIIT as the primary exercise intervention, although a few trials incorporated additional rehabilitation components such as respiratory muscle training or resistance exercises as part of multimodal perioperative programs. Specific data are presented in Table 1. The methodological quality of the included RCTs was evaluated using the Cochrane risk-of-bias tool. The majority of studies showed a low risk of bias in key domains such as random sequence generation and incomplete outcome data. However, blinding of participants and personnel was generally not feasible given the nature of the exercise interventions. Detailed assessments are shown in Figures 2A,B.

**Table 1:** Basic characteristics of the included studies.

Author	Year	Country	Sample size		Intervention period	Endpoint
			Patients in the HIIT group	Patients in the control group		
Fang et al. (17)	2013	China	22	22	Pre-operation	Complication, postoperative length of stay
Edvardsen et al. (18)	2015	Norway	30	31	Post-operation	Exercise capacity, pulmonary function, quality of life
Wu et al. (19)	2015	China	29	29	Pre-operation	Complication, postoperative length of stay
Cavalheri et al. (20)	2017	Australia	9	8	Post-operation	Exercise capacity, quality of life
Licker et al. (21)	2017	Switzerland	74	77	Pre-operation	Complication
Liu et al. (22)	2017	China	34	34	Pre-operation	Complication, pulmonary function
Bhatia and Kayser (23)	2019	Switzerland	74	77	Pre-operation	Exercise capacity
Messaggi-Sartor et al. (24)	2019	Spain	16	21	Post-operation	Exercise capacity, respiratory muscle strength
Liu et al. (25)	2020	China	37	36	Pre-operation	Complication, postoperative length of stay
Gao et al. (26)	2022	China	89	89	Post-operation	Pulmonary function, exercise capacity, quality of life, emotional performance
Huang et al. (28)	2025	China	30	30	Pre-operation	Complication, length of stay

HIIT: high-intensity interval training.

### Effect of HIIT on the primary clinical outcomes among surgical lung cancer patients

**Figure 3:**
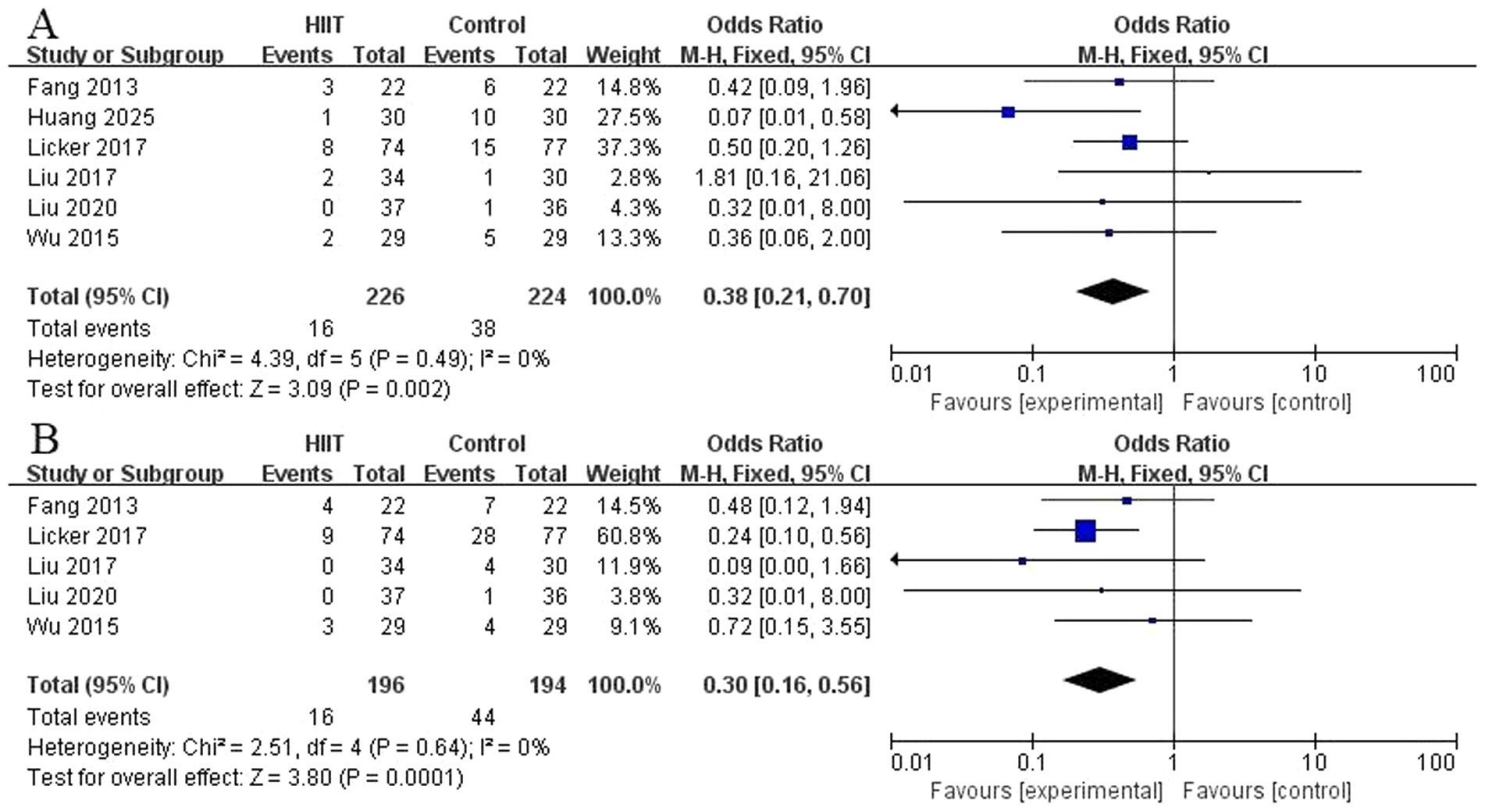
Effect of high-intensity interval training on the risk of pulmonary infection **(A)** and pulmonary atelectasis **(B)** among surgical lung cancer patients.

In total, six studies explored the impact of HIIT on the risk of postoperative complications in lung cancer patients undergoing surgery (17, 19, 21, 22, 25, 28). Pooled results revealed that HIIT significantly decreased the risk of postoperative pulmonary infection (OR = 0.38, 95% CI: 0.21 – 0.70, *p* = 0.002; I^2^ = 0%, *p* = 0.49) (Figure 3A) and pulmonary atelectasis (OR = 0.30, 95% CI: 0.16 ~ 0.56, *p <* 0.001; I^2^ = 0%, *p* = 0.64) (Figure 3B; Table 2).

**Table 2:** Results of meta-analysis for primary outcomes.

Items	Number of studies	OR	95% CI	*p*-value	I^2^ (%)	*p*-value for heterogeneity	Certainty of evidence
Pulmonary infection	6	0.38	0.21 ~ 0.70	0.002*	0	0.49	Moderate
Pulmonary atelectasis	5	0.30	0.16 ~ 0.56	*<* 0.001*	0	0.64	Moderate
Arrhythmia	4	0.67	0.36 ~ 1.25	0.21	45	0.14	Moderate
30-day mortality	3	0.56	0.12 ~ 2.67	0.47	0	0.35	Moderate
Respiratory failure	2	0.40	0.15 ~ 1.06	0.06	0	0.65	Low
Cardiovascular complication	2	1.44	0.64 ~ 3.22	0.37	0	0.96	Low
Grade 2 + complication	1	0.75	0.18 ~ 3.06	0.69	-	-	Low
Cardiopulmonary complication	1	0.54	0.15 ~ 1.92	0.34	-	-	Low
Bronchopleural fistula	1	1.04	0.20 ~ 5.34	0.96	-	-	Low

OR: odds ratio; CI: confidence interval; *: significant statistical difference.

However, based on current available evidence, no significant association was observed between the HIIT intervention and risk of postoperative arrhythmia (OR = 0.67, 95% CI: 0.36 ~ 1.25, *p* = 0.21) (Supplementary Figure 1A), 30-day mortality (OR = 0.56, 95% CI: 0.12 ~ 2.67, *p* = 0.47) (Supplementary Figure 1B), respiratory failure (OR = 0.40, 95% CI: 0.15 ~ 1.06, *p* = 0.06) (Supplementary Figure 1C), cardiovascular complication (OR = 1.44, 95% CI: 0.64 ~ 3.22, *p* = 0.37) (Supplementary Figure 1D), grade 2 + complication (OR = 0.75, 95% CI: 0.18 ~ 3.06, *p* = 0.69), cardiopulmonary complication (OR = 0.54, 95% CI: 0.15 ~ 1.92, *p* = 0.34), or bronchopleural fistula (OR = 1.04, 95% CI: 0.20 ~ 5.34, *p* = 0.96) (Table 2).

According to the GRADE assessment, the certainty of evidence ranged from low to moderate across outcomes. Moderate-certainty evidence was observed for pulmonary infection, atelectasis, arrhythmia, and 30-day mortality.

### Effect of HIIT on the secondary clinical outcomes among surgical lung cancer patients

**Figure 4:**
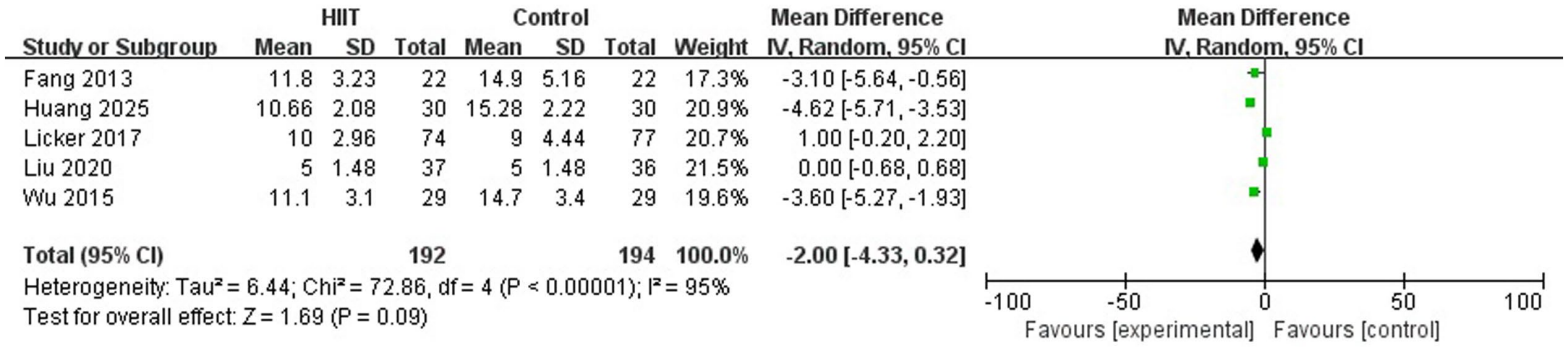
Effect of high-intensity interval training on the postoperative length of stay among surgical lung cancer patients.

**Figure 5:**
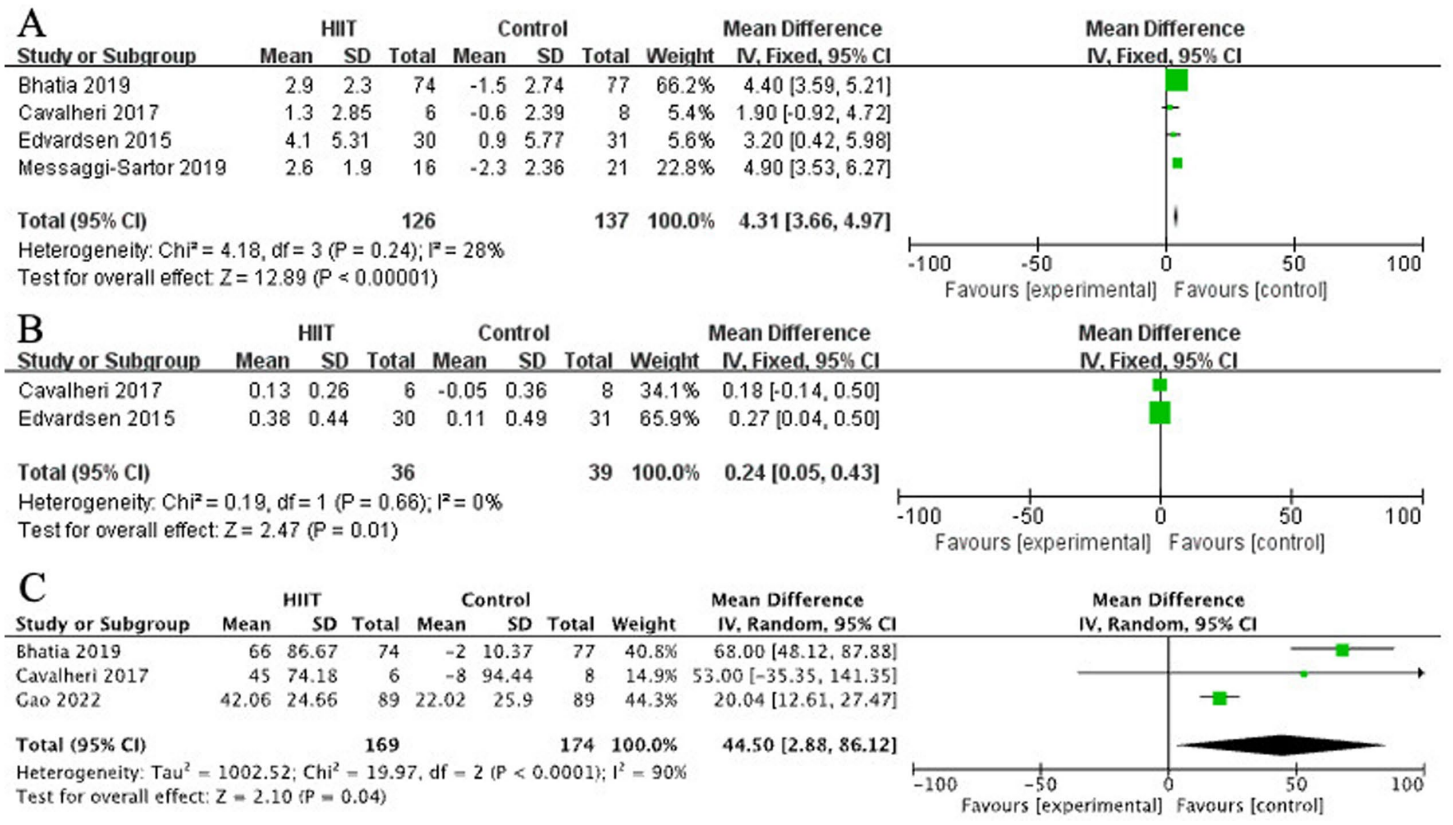
Effect of high-intensity interval training on the peak oxygen uptake (mL/kg/min) **(A,B)** and 6-min walk distance **(C)** among surgical lung cancer patients.

**Figure 6:**
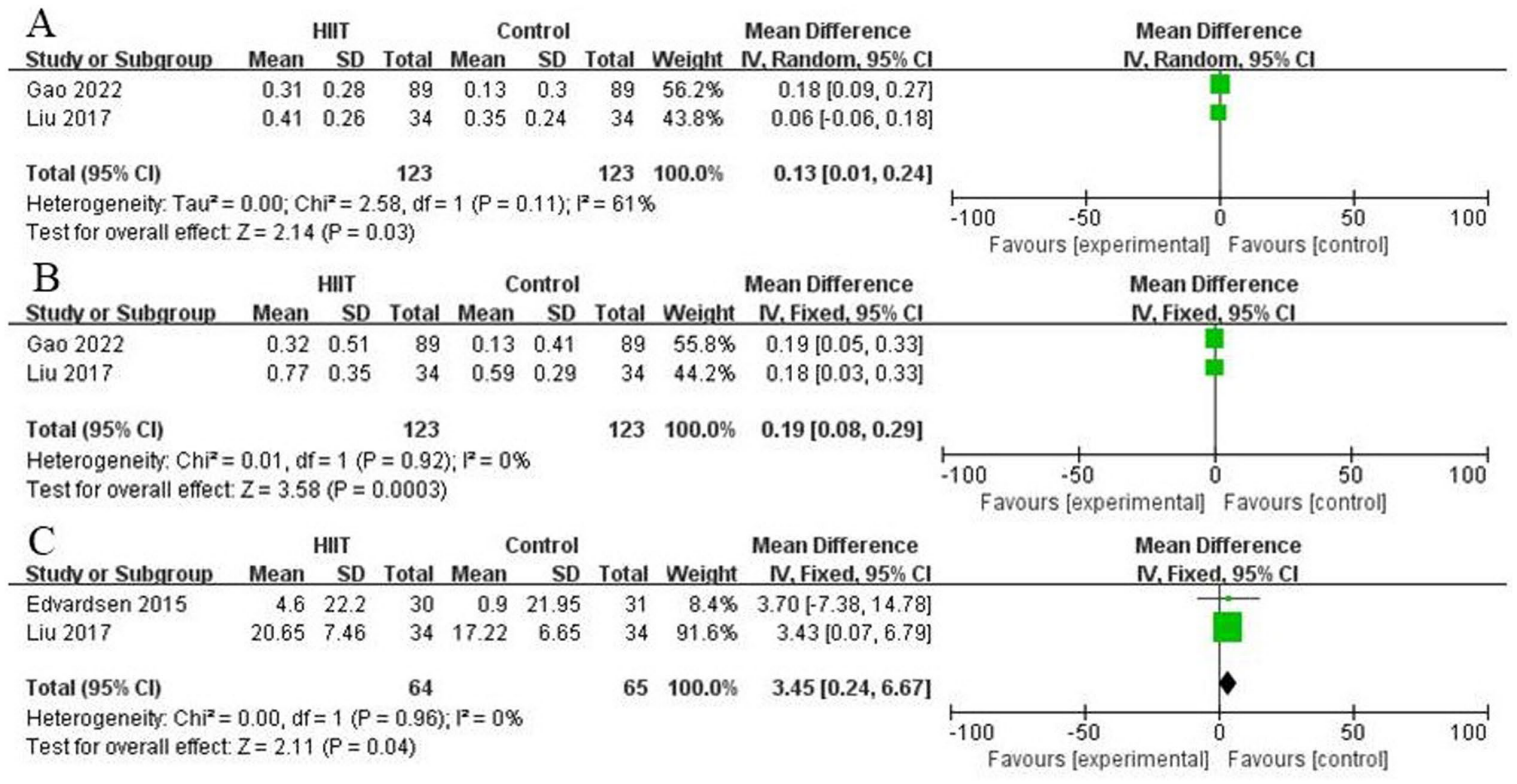
Effect of high-intensity interval training on the forced vital capacity **(A)**, forced vital capacity **(B)**, and maximum voluntary ventilation% **(C)** among surgical lung cancer patients.

For postoperative LOS, no significant impact of HIIT was detected on the postoperative LOS (MD = −2.00d, 95% CI: −4.33 ~ 0.32d, *p* = 0.09; I^2^ = 95%, *p <* 0.001) (Figure 4). However, HIIT played a role in improving the exercise capacity, as reflected by increased peak oxygen uptake (MD = 4.31 mL/kg/min, 95% CI: 3.66 ~ 4.97 mL/kg/min, *p <* 0.001; I^2^ = 28%, *p* = 0.24) (Figure 5A) (MD = 0.24 L/min, 95% CI: 0.05 ~ 0.43 L/min, *p* = 0.01; I^2^ = 0%, *p* = 0.66) (Figure 5B) and 6MWD (MD = 44.50 m, 95% CI: 2.88 ~ 86.12 m, *p* = 0.04; I^2^ = 90%, *p <* 0.001) (Figure 5C). In addition, it was shown that HIIT significantly improved pulmonary function among surgical lung cancer patients with increased FEV1 (MD = 0.13 L, 95% CI: 0.01 ~ 0.24 L, *p* = 0.03; I^2^ = 61%, *p* = 0.11) (Figure 6A), FVC (MD = 0.19 L, 95% CI: 0.08 ~ 0.29 L, *p <* 0.001; I^2^ = 0%, *p* = 0.92) (Figure 6B), and MVV (MD = 3.19 L/min, 95% CI: 1.18 ~ 5.20 L/min, *p* = 0.002) (MD = 3.45, 95% CI: 0.24 ~ 6.67%, *p* = 0.04; I^2^ = 0%, *p* = 0.96) (Figure 6C; Table 3).

**Table 3:** Results of meta-analysis for secondary outcomes.

Items	Number of studies	MD	95% CI	*p*-value	I^2^ (%)	*p*-value for heterogeneity	Certainty of evidence
Postoperative length of stay (d)	5	−2.00	−4.33 ~ 0.32	0.09	95	*<* 0.001	Low
Exercise capacity							
Peak oxygen uptake (mL/kg/min)	4	4.31	3.66 ~ 4.97	*<* 0.001*	28	0.24	Moderate
Peak oxygen uptake (L/min)	2	0.24	0.05 ~ 0.43	0.01*	0	0.66	Low
Peak oxygen uptake (% predicted)	1	10.00	−9.01 ~ 29.01	0.30	-	-	Low
6MWD (m)	3	44.50	2.88 ~ 86.12	0.04*	90	*<* 0.001	Low
6MWD (% predicted)	1	9.00	−2.64 ~ 20.64	0.13	-	-	Low
Pulmonary function							
FEV1 (L)	2	0.13	0.01 ~ 0.24	0.03*	61	0.11	Low
FEV1 (%)	1	0.70	−8.80 ~ 10.20	0.89	-	-	Low
FVC (L)	2	0.19	0.08 ~ 0.29	*<* 0.001*	0	0.92	Low
MVV (L/min)	1	3.19	1.18 ~ 5.20	0.002*	-	-	Low
MVV (%)	2	3.45	0.24 ~ 6.67	0.04*	0	0.96	Low
Quality of life							
SF-36 (overall score)	1	8.32	4.31 ~ 12.33	*<* 0.001*	-	-	Low
SF-36 (physical component)	2	7.04	2.96 ~ 11.13	*<* 0.001*	2	0.31	Low
SF-36 (mental component)	2	−0.13	−16.67 ~ 16.41	0.99	89	0.003	Low
EORTC LC13	1	−11.00	−23.55 ~ 1.55	0.09	-	-	Low
Respiratory muscle strength							
MIP (% predicted)	1	16.10	−1.99 ~ 34.19	0.08	-	-	Low
MEP (% predicted)	1	7.30	−5.50 ~ 20.10	0.26	-	-	Low
Emotional performance							
SAS score	1	−6.90	−10.28 ~ −3.52	*<* 0.001*	-	-	Low
FoP-Q-SF score	1	−5.12	−8.40 ~ −1.84	0.002*	-	-	Low

MD: mean difference; CI: confidence interval; 6MWD: 6-min walk distance; FEV1: forced expiratory volume in 1 s; FVC: forced vital capacity; MVV: maximum voluntary ventilation; SF-36: Medical Outcomes Study Short-Form 36 general health survey; EORTC LC13: Lung Cancer subscale of the European Organization for Research and Treatment of Cancer, Quality of Life Questionnaire Core 30; MIP: expiratory pressure; MEP: maximal expiratory pressure; SAS: Self-rating Anxiety Scale; FoP-Q-SF: Fear of Progression Questionnaire-Short Form; *: significant statistical difference.

Meanwhile, pooled results indicated that HIIT could improve the quality of life assessed by the SF-36 (overall score: MD = 8.32, 95% CI: 4.31 ~ 12.33, *p <* 0.001) (physical component: MD = 7.04, 95% CI: 2.96 ~ 11.13, *p <* 0.001; I^2^ = 2%, *p* = 0.31) (Supplementary Figure 2A) and emotional outcomes assessed by the SAS score (MD = −6.90, 95% CI: −10.28 ~ −3.52, *p <* 0.001) and FoP-Q-SF score (MD = −5.12, 95% CI: −8.40 ~ −1.84, *p* = 0.002). However, HIIT did not show a significant role in improving the respiratory muscle strength (MIP % predicted: MD = 16.10, 95% CI: −1.99 ~ 34.19%, *p* = 0.08; MEP % predicted: MD = 7.30, 95% CI: −5.50 ~ 20.10%, *p* = 0.26) (Table 3).

According to the GRADE assessment, the certainty of evidence ranged from low to moderate across outcomes. Moderate-certainty evidence was only observed for peak oxygen uptake (mL/kg/min).

## Discussion

This meta-analysis systematically evaluated the effects of HIIT on clinical outcomes in lung cancer patients undergoing surgery. The pooled results revealed that HIIT was associated with a lower risk of postoperative pulmonary complications, particularly pulmonary infection and atelectasis, while improving pulmonary function, exercise capacity, quality of life, and emotional status. These findings provide supportive evidence for the clinical value of HIIT as an effective perioperative rehabilitation strategy for enhancing recovery and functional outcomes in surgical lung cancer patients. Our meta-analysis demonstrated that HIIT may also reduce the incidence of postoperative pulmonary complications, including pulmonary infection and atelectasis, in patients undergoing lung cancer surgery. These protective effects may be attributed to multiple mechanisms. Postoperative pulmonary infection and atelectasis are primarily caused by impaired ventilation, mucus retention, and reduced cough effectiveness after thoracic surgery. Therefore, interventions that improve airway clearance and ventilatory capacity may directly reduce these complications. HIIT has been shown to enhance respiratory muscle strength, increase mucociliary clearance, and improve alveolar ventilation, which collectively reduce postoperative pulmonary stasis and secretion retention (29–31). Enhanced mucociliary clearance facilitates the removal of airway secretions and pathogens, thereby lowering the risk of postoperative pulmonary infection. At the same time, improved respiratory muscle performance and tidal ventilation increase alveolar recruitment and reduce the likelihood of alveolar collapse, which is a major contributor to postoperative atelectasis. Additionally, preoperative HIIT enhances cardiopulmonary reserve and oxygen transport capacity, facilitating better tolerance to anesthesia and surgical stress (32). These cardiopulmonary adaptations may help maintain adequate postoperative ventilation–perfusion matching and oxygen delivery, thereby further preventing hypoventilation-related complications such as atelectasis and infection. Improved ventilation–perfusion matching and strengthened cough efficacy can further reduce alveolar collapse and infection risk (33, 34). Furthermore, regular high-intensity training reduces systemic inflammation and oxidative stress, both of which are implicated in the pathogenesis of postoperative pulmonary complications (35). Reduced systemic inflammation may attenuate postoperative inflammatory responses in lung tissue, thereby limiting airway edema and secretion accumulation that contribute to pulmonary infection and atelectasis. Collectively, these physiological adaptations may underlie the reduced risk of pulmonary infection and atelectasis observed in surgical lung cancer patients undergoing HIIT.

In contrast, no significant difference in cardiac complications was observed between the two groups. This finding may be explained by the multifactorial nature of postoperative cardiac events, which are often influenced by preexisting cardiovascular comorbidities, intraoperative hemodynamic fluctuations, anesthetic management, and perioperative cardiac risk profiles rather than by respiratory function alone. Although improved perioperative respiratory status may contribute to better overall recovery, it may not be sufficient to significantly modify the incidence of cardiac complications, especially in patients with established cardiovascular risk factors. Additionally, the relatively small number of included studies and events may have limited the statistical power to detect potential differences between the groups.

In addition to reducing postoperative complications, HIIT significantly improves exercise capacity, pulmonary function, and emotional outcomes. HIIT effectively promotes mitochondrial biogenesis, enhances skeletal muscle oxidative enzyme activity, and increases maximal oxygen uptake (VO₂peak), resulting in superior endurance and aerobic capacity (35, 36). The interval nature of HIIT allows patients to perform higher-intensity work with reduced fatigue, leading to greater cardiopulmonary adaptations than moderate continuous training (8). Improved pulmonary function, as indicated by increased FEV₁ and FVC, may reflect enhanced respiratory muscle coordination and reduced postoperative restrictive changes (37, 38). Moreover, exercise training exerts positive psychological effects by alleviating anxiety, depression, and fear of disease progression through neuroendocrine modulation and improved self-efficacy (39). Collectively, these findings suggest that HIIT may provide both physiological and psychological benefits that support overall recovery in lung cancer patients.

From a clinical perspective, HIIT may serve as an effective and feasible component of perioperative rehabilitation programs for lung cancer patients. It may be particularly beneficial for high-risk individuals, such as those with reduced baseline exercise capacity, impaired pulmonary function, or advanced age (40). Evidence has suggested that preoperative programs lasting 2–4 weeks, involving three to five sessions per week of 20–30 min each, can yield measurable improvements in cardiorespiratory fitness and postoperative outcomes (21, 41, 42). A typical HIIT protocol includes alternating 1–4 min of high-intensity cycling or treadmill exercise (at 80–100% of peak work rate) with equal or longer recovery intervals (21, 23). Individualized supervision by physiotherapists is recommended to ensure safety and optimize compliance. Integration of HIIT into enhanced recovery after surgery (ERAS) pathways may further accelerate functional recovery and reduce hospital stay (43, 44). Future studies should focus on protocol standardization, long-term adherence, and cost-effectiveness to guide broader clinical implementation.

Notably, substantial heterogeneity was observed for certain outcomes, particularly postoperative LOS and 6MWD. Although potential sources of heterogeneity may include differences in intervention timing (preoperative versus postoperative), training duration, training intensity, frequency, surgical approach, and discharge criteria, formal subgroup or meta-regression analyses were not feasible. For the majority of outcomes, only a few studies (generally 1–3, and at most 5–6) were available. Conducting subgroup analyses under these conditions would have resulted in very few studies per subgroup, substantially reducing statistical power and potentially generating unreliable estimates. Therefore, the pooled results for outcomes with high heterogeneity should be interpreted with caution. Future large-scale, well-designed randomized controlled trials with standardized intervention protocols are needed to better explore the sources of heterogeneity.

There are some limitations in this meta-analysis. First, there were obvious differences in the intervention plans and timing of HIIT across studies. In the future, larger-sample studies are needed to better standardize HIIT intervention protocols. Second, we mainly focused on short-term clinical outcomes such as postoperative complications and exercise capacity; long-term clinical outcomes, such as survival, should be further investigated. Third, significant heterogeneity was observed for some outcomes. Due to the limited number of included studies per outcome, subgroup or sensitivity analyses based on clinically relevant factors (e.g., intervention timing, training characteristics, or surgical approach) were not performed. Consequently, the pooled estimates for outcomes with substantial heterogeneity should be interpreted cautiously. Fourth, the number of included studies for each outcome was relatively small, with most pooled analyses including only 1–3 studies and at most 5–6 studies. Although 7 of the 11 included publications reported preoperative rehabilitation, we did not perform a subgroup analysis based on preoperative rehabilitation status. Given the limited number of studies per outcome, conducting such subgroup analyses would have resulted in very small sample sizes within subgroups, thereby substantially reducing statistical power and the reliability of the findings. Therefore, the results should be interpreted with caution, and future studies with larger sample sizes are needed to further explore the potential impact of preoperative rehabilitation on outcomes. Fifth, although HIIT was the core component of the intervention in all included studies, several trials incorporated additional rehabilitation components, such as resistance training or respiratory exercises, as part of multimodal prehabilitation programs. This may introduce clinical heterogeneity, and the observed benefits cannot be entirely attributed to HIIT alone. Due to the limited number of available studies, subgroup or sensitivity analyses separating pure HIIT interventions from combined rehabilitation programs were not feasible. Therefore, the pooled effects should be interpreted with caution, and future randomized controlled trials are warranted to clarify the independent effect of HIIT.

## Conclusion

This meta-analysis demonstrated that HIIT may provide clinical benefits for patients with lung cancer undergoing surgery. Specifically, HIIT may be associated with a reduced incidence of postoperative pulmonary complications, including pulmonary infection and atelectasis, and improved pulmonary function, exercise capacity, quality of life, and emotional wellbeing. These findings suggest that incorporating HIIT into perioperative rehabilitation programs may enhance recovery and overall outcomes in surgical lung cancer patients. However, given the heterogeneity in exercise protocols and sample sizes among the included trials, further large-scale, multicenter randomized controlled studies are warranted to confirm these results and to optimize HIIT protocols tailored for this specific patient population.

## Data Availability

The original contributions presented in the study are included in the article/Supplementary material, further inquiries can be directed to the corresponding author.
