# Supplementary material for: Effect of high-intensity interval training on clinical outcomes in lung cancer patients undergoing surgery: a meta-analysis based on randomized controlled trials
Source: Front Med (Lausanne). 2026 Jul 10;13:1868572. doi: 10.3389/fmed.2026.1868572 (PMC13395777; doi:10.3389/fmed.2026.1868572)
Supplement: Supplementary file 1 [file Presentation_1.PPTX]

## Slide 1
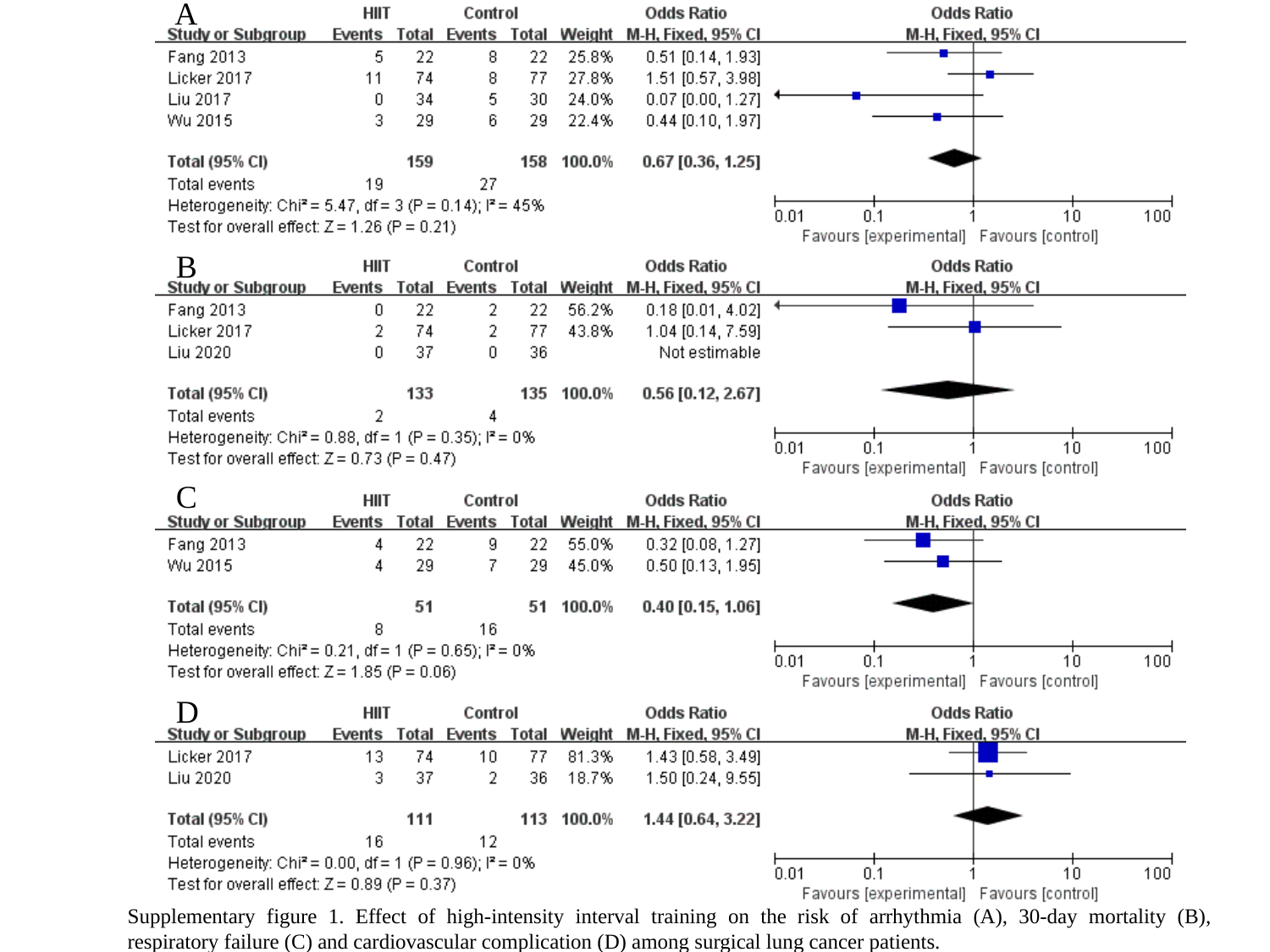

A
B
C
D
Supplementary figure 1. Effect of high-intensity interval training on the risk of arrhythmia (A), 30-day mortality (B), respiratory failure (C) and cardiovascular complication (D) among surgical lung cancer patients.
