# Supplementary material for: Effect of high-intensity interval training on clinical outcomes in lung cancer patients undergoing surgery: a meta-analysis based on randomized controlled trials
Source: Front Med (Lausanne). 2026 Jul 10;13:1868572. doi: 10.3389/fmed.2026.1868572 (PMC13395777; doi:10.3389/fmed.2026.1868572)
Supplement: Supplementary file 2 [file Presentation_2.PPTX]

## Slide 1
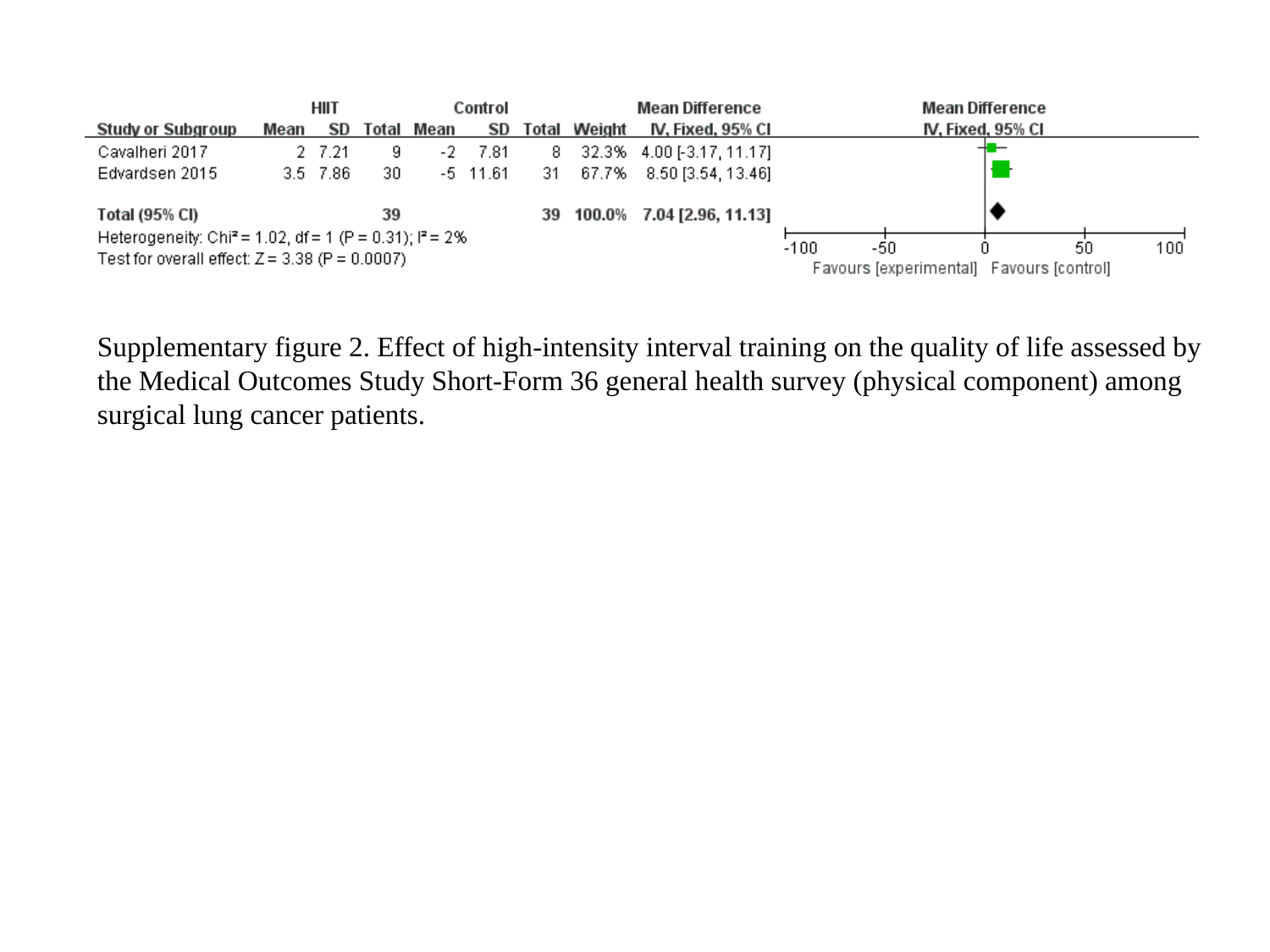

Supplementary figure 2. Effect of high-intensity interval training on the quality of life assessed by the Medical Outcomes Study Short-Form 36 general health survey (physical component) among surgical lung cancer patients.
