# Supplementary material for: Effect of high-intensity interval training on clinical outcomes in lung cancer patients undergoing surgery: a meta-analysis based on randomized controlled trials
Source: Front Med (Lausanne). 2026 Jul 10;13:1868572. doi: 10.3389/fmed.2026.1868572 (PMC13395777; doi:10.3389/fmed.2026.1868572)
Supplement: Supplementary file 3 [file Data_Sheet_1.DOCX]

Supplementary file 1. Specific search strategies in individual databases.

**Pubmed:**

#1 "High-Intensity Interval Training"[Mesh]

OR "high intensity interval training"[Title/Abstract]

OR HIIT[Title/Abstract]

#2 "Lung Neoplasms"[Mesh]

OR lung cancer[Title/Abstract]

OR pulmonary cancer[Title/Abstract]

OR lung tumor[Title/Abstract]

OR lung tumour[Title/Abstract]

OR lung neoplasm*[Title/Abstract]

OR pulmonary neoplasm*[Title/Abstract]

OR carcinoma[Title/Abstract]

#3 #1 AND #2

Filters applied: None at search stage

Language restriction applied during screening: English or Chinese

**CNKI:**

("高强度间歇训练" OR "高强度间歇运动" OR HIIT)

AND

("肺癌" OR "肺肿瘤" OR "肺部肿瘤")

**WOS:**

TS = (

("high intensity interval training" OR HIIT)

AND

(lung OR pulmonary)

AND

(cancer OR carcinoma OR neoplasm* OR tumor OR tumour)

)

Indexes searched:

SCI-EXPANDED

No document type restriction applied during search stage.

Language restriction (English or Chinese) applied during screening.

**Cochrane library:**

("high intensity interval training" OR HIIT)

AND

(lung OR pulmonary)

AND

(cancer OR carcinoma OR neoplasm* OR tumor OR tumour)
